# Angioplasty with the scepter C dual lumen balloon catheter and postprocedural result evaluation in patients with subarachnoid hemorrhage related vasospasms

**DOI:** 10.1186/s12883-020-01792-3

**Published:** 2020-06-29

**Authors:** Ioannis Tsogkas, Vesna Malinova, Katharina Schregel, Dorothee Mielke, Daniel Behme, Veit Rohde, Michael Knauth, Marios-Nikos Psychogios

**Affiliations:** 1 Department of Neuroradiology, University Medical Center Goettingen, Robert-Koch-Str. 40, 37075 Gottingen, Germany; 2 Department of Neuroradiology, Clinic of Radiology & Nuclear Medicine, University Hospital Basel, Petersgraben 4, 4031 Basel, Basel Switzerland; 3 Department of Neurosurgery, University Medical Center Goettingen, Robert-Koch-Str. 40, 37075 Goettingen, Lower Saxony Germany; 4 Department of Neuroradiology, Heidelberg University Hospital, Heidelberg, Germany

**Keywords:** Vasospasms, Angioplasty, Balloon-catheter, Subarachnoid hemorrhage, Scepter C balloon catheter, iFlow tool

## Abstract

**Background:**

Delayed cerebral ischemia is one of the leading causes of death and disability in patients with subarachnoid hemorrhage (SAH). Transluminal balloon angioplasty (TBA) is a therapeutic option for vasospasms affecting proximal intracranial arteries.

**Methods:**

Aim of this study was to report our experience using the Scepter C balloon catheter in the treatment of cerebral vasospasms due to SAH and evaluate the postprocedural result with the iFlow tool. We reviewed cases of patients treated at our hospital from 2014 to 2018. Patients were screened with transcranial doppler sonography (TCD) and multimodal computed tomography. In case of significant vasospasms, patients were transferred to the angiography suite and treated. We used the iFlow tool to quantify and evaluate the angiographic results by measuring and comparing peak density values on angiograms before and after the mechanical dilation.

**Results:**

The use of the Scepter C balloon catheter was feasible in all cases. Vasospasms of the anterior cerebral artery were treated in ten cases. We didn’t observe complications or vasospasm recurrences of the treated arteries. The temporal difference between distal vessels and the proximal reference vessel was significantly reduced from a mean of 53%, prior to dilatation, to 26% after the treatment. The difference between pre-dilatation and post-dilatation values was statistically significant for the anterior circulation at the proximal as well as at the distal vessels.

**Conclusions:**

We successfully treated endovascularly patients suffering from cerebral vasospasms refractory to medical treatment using the Scepter C balloon catheter. We didn’t observe any complications. The therapeutic effect could be easily and reliably assessed with the iFlow tool.

## Background

Cerebral vasospasm is defined as the narrowing of large or small intracranial arteries after a subarachnoid hemorrhage (SAH). Angiographic vasospasms are very common in patients with a ruptured intracranial aneurysm, with an incidence of 50–90% [1, 2]. Delayed ischemic neurological deficit or symptomatic vasospasm is one of the leading causes of death and disability in these patients and occurs in 20–25% of them [3, 4]. Although the mechanism of nimodipine’s beneficial effect is not completely clear, in eight randomized clinical trials nimodipine has shown to have a statistically significant effect on the outcome [5–11]. Thus, it is routinely used as a preventive measure as well as a treatment for cerebral vasospasms.

Transluminal balloon angioplasty (TBA) is a therapeutic option for symptomatic vasospasms in proximal vessels after SAH. The results of a recent international survey by Hollingworth et al. demonstrated that 83% of non-United States (U.S.) physicians and 91% of U.S. physicians performed angioplasty for treatment of vasospasms. Despite the universally accepted efficacy of angioplasty in patients with cerebral vasospasm, only 3% of U.S. responders and 6% of non-U.S. responders used it as a first-line endovascular therapy [12].

Multiple studies have reported a reversal of neurological deficits after angioplasty in 30–70% of patients in whom the medical treatment failed [13–16]. However, complications including wire perforation, vessel dissection and ischemic stroke have been reported. Nevertheless, endovascular therapies, techniques as well as materials have been evolved since the first report of balloon catheter usage [17].

We report our experience with the dual lumen, compliant balloon catheter in the endovascular treatment of vasospasms after SAH. We used a commercially available software to retrospectively assess the treatment efficacy of angioplasty comparing time-density curves before and after treatment.

## Methods

### Patient selection

We reviewed patients from our center from 2014 to 2018 who suffered from vasospasms after cerebral aneurysm rupture and were treated with the Scepter C balloon catheter. All SAH-patients underwent screening with transcranial doppler sonography (TCD) on a daily basis. A mean blood flow velocity (BFV) > 120 cm/sec was defined as TCD-confirmed vasospasm [18]. We performed computed tomography angiography (CTA) and computed tomography perfusion (CTP) routinely in the acute phase (3rd - 5th day after aSAH) and/or in case of clinical deterioration. In intubated patients, we performed a CT-imaging on the 3rd and 7th day or in the event of significantly elevated TCD velocities (higher as 120 cm/s or increase of 50 cm/sec within 24 h).

### Angioplasty technique

In the case of perfusion deficits, notable acceleration of the TCD velocities or clinical vasospasms, and after exclusion of a large cerebral infarct in CT, patients were transferred to the angiography suite. An experienced neuroradiologist (more than 5 years of experience) performed a TBA via a transfemoral approach in cases of significant vasospasms of proximal vessels. We performed a diagnostic angiography after the placement of a 5F or 6F sheath in the femoral artery to elucidate which vessels should be treated. The images were acquired after injection of 8 mL of contrast agent (Imeron 400, Bracco, Konstanz, Germany) manually by hand at a flow rate of 4 mL/s for 2 s.

**Fig. 1:**
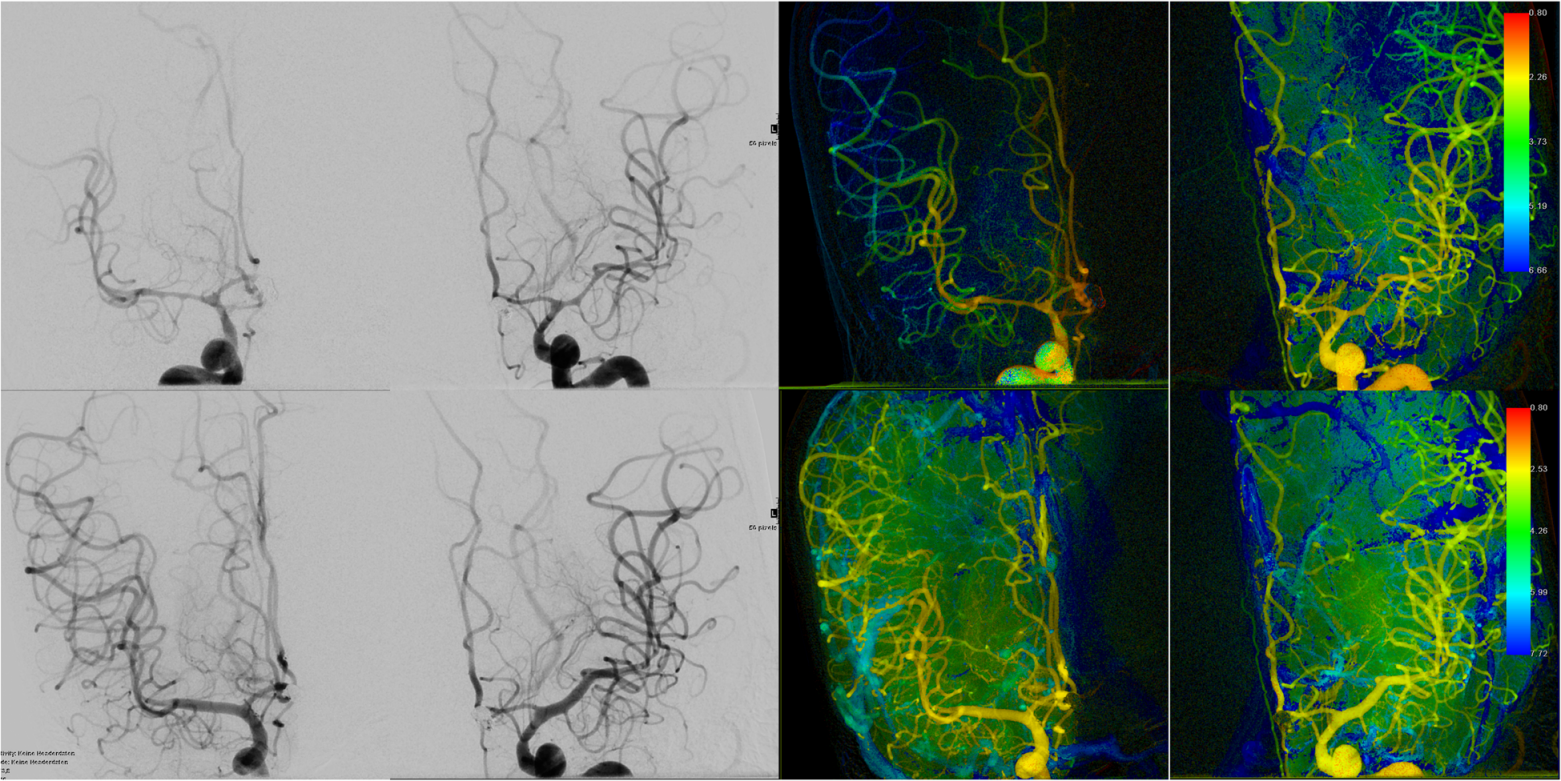
Angiographic and color-coded images processed with the iFlow tool of a 47-year-old female patient. Rupture and immediate coiling of a ACom-aneurysm. After 4 days significant vasospasms of the left and right MCA and Carotis-T arteries are depicted. Distal ICA and right M1 segment, left M1 and M2 were successfully treated with the Scepter C balloon catheter. An angioplasty of ACA wasn’t initially performed because of the recent coiling. Temporal delay between proximal and distal segments was significantly reduced after treatment

**Fig. 2:**
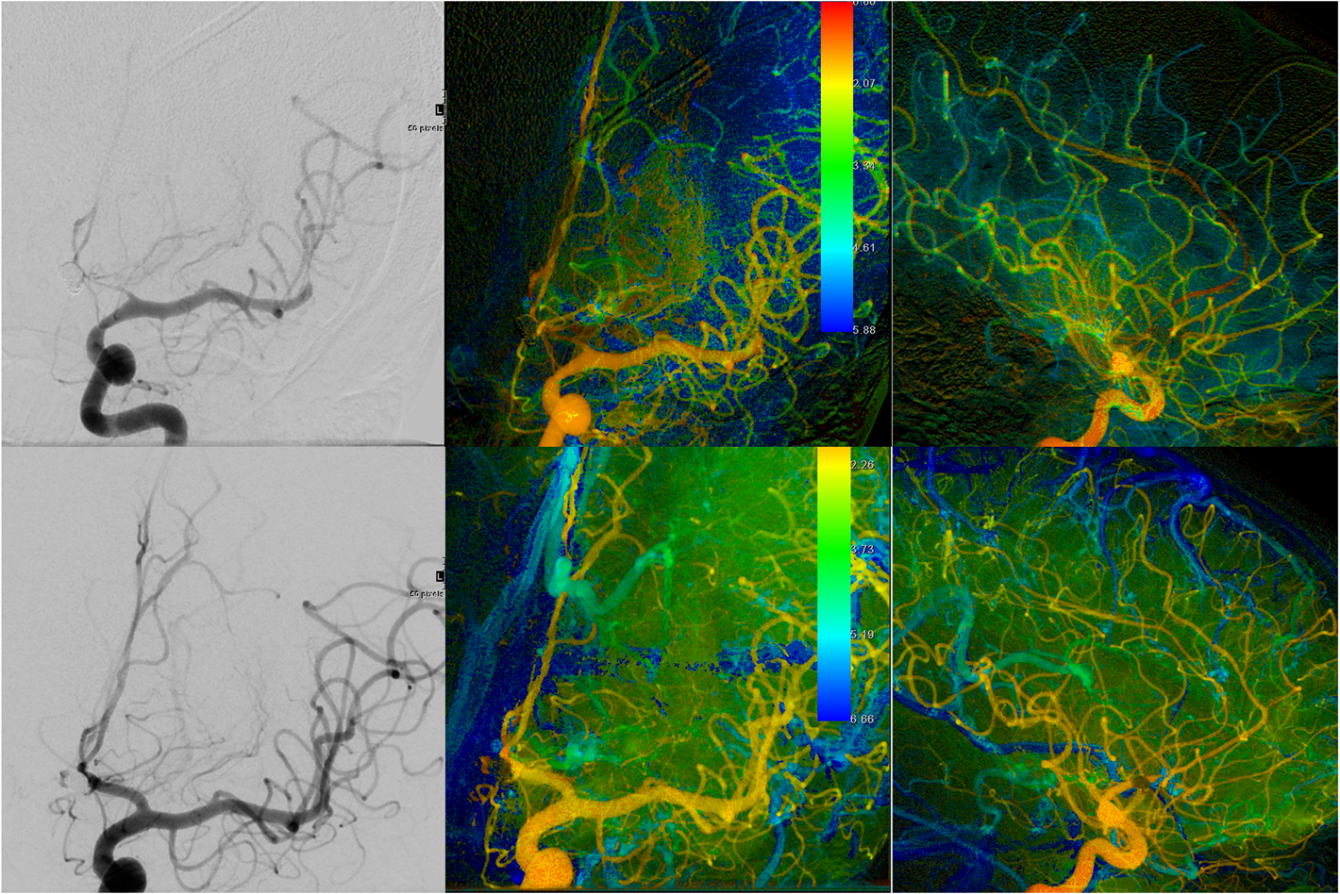
Angiographic and color-coded (pa und lateral) images processed with the iFlow tool of the same patient. Rupture and immediate coilembolization of a ACom-aneurysm. Three days after the first angioplasty, treatment with PTA of the left ACA because of persistent significant perfusion deficit

If a TBA was necessary, we placed proximally a heparinized saline-flushed 5 or 6 French guide catheter and dilated the vasospastic vessels with the Scepter C balloon catheter (Microvention Tustin, California, USA) (Figs. 1 and 2). The use of additional intra-arterial nimodipine was based on the discretion of the interventionalist and was administered either before or after the angioplasty. All interventions were performed under general anesthesia; patients without contraindications were extubated directly after the procedure in the angiography suite and underwent neurologic examination. Indicators for complications related to the procedure were the angiographic images after the angioplasty and the clinical examination of the patients, who were intubated only for the procedure and extubated immediately after that. According to our procedural standard patients underwent a flat-detector computed tomography at the end of the intervention in order to exclude a new bleeding.

We transferred patients to the intensive care unit, where they were treated for 14 days or longer if needed. We were able to acquire the follow-up mRS at 3 months in 11 patients.

### Analysis

We retrospectively analyzed our data and postprocessed hemodynamic parameters of angiograms before and after the TBA in a dedicated image reconstruction workstation (syngo X Workplace; Siemens Healthcare, Forchheim, Germany) using quantitative DSA with syngo iFlow software (Siemens). This software provides a color-coded single image by calculating and displaying time-intensity curves for each image pixel [19]. We placed 6 regions of interest (ROI) in two projection angiograms in order to assess the hemodynamic parameters in proximal arteries (middle cerebral artery [MCA] and anterior cerebral artery [ACA], M1 and A1 segment), as well as in distal arteries (postcentral branch of MCA and pericallosal artery, M4- and A2 segment). For the posterior circulation, we used 3 ROIs in the distal basilar artery, in the proximal posterior cerebral artery (P1 segment) and in the distal posterior cerebral artery (P2 segment). Reference vessels were the extradural or cavernous internal carotid artery for the anterior circulation and the extradural vertebral artery for the posterior circulation where no vasospasms were noticed. The hemodynamic parameters were processed with the aforementioned software and were analyzed with the MedCalc statistical package (MedCalc Statistical Software version 17.7, MedCalc Software bvba, Ostend, Belgium; http://www.medcalc.org; 2017). The significance level for all tests was set to α = 0.05.

## Results

We treated nineteen SAH-patients (average age 51, ±11.6 years, eleven women) suffering from SAH-related vasospasms with the Scepter C balloon catheter. Because of motion artifacts the angiograms of two patients could not be processed with the abovementioned software and these patients were excluded from the evaluation. We retrospectively analyzed 22 cases (procedures) of the seventeen included patients.

Three patients suffered from rupture of an aneurysm of the intradural internal carotid artery (6 cases), two patients from rupture of an aneurysm of the MCA (2 cases), five patients suffered from the rupture of an aneurysm of the anterior communicating artery (6 angioplasty cases), two patients from rupture of an aneurysm of the basilar artery (2 cases), two patients from rupture of an aneurysm of the posterior inferior cerebellar artery (3 cases), and one patient suffered from the rupture of an aneurysm of the vertebral artery (1 case). One patient suffered from SAH after the endovascular therapy of an aneurysm of the basilar artery (coil-embolization) and one patient suffered from SAH and vasospasms but no aneurysm or vessel malformation were detected on initial or follow-up digital subtraction angiography (DSA) (Table 1).

**Table 1:** Baseline characteristics, treatment times, aneurysm location, SAB classification, balloon size, use of intraarterial medical treatment, dilated vessels, complications and treatment of the ruptured aneurysm. Baseline Characteristics of patients

Pt/Case	Days to dilatation	Aneurysm location	Grad Fischer, Hunt-Hess	Balloon Size	Intraarterial medical vasodilatation	Dilatated Vessels	Complic.	Treatment
1/1	7	PICA	III, IV	4 × 15	Yes	BA, LV, RCI, LCI, RM1, LM1	No	Coiling
2/2	8	ACOM	III, I	4 × 20	Yes	RA1, RM1, LM1, LA1	No	Coiling and Clipping
3/3	6	ACOM	IV, V	4 × 15	Yes	RCa-T, RM1, LCa-T, LM1, LV	No	Coiling
3/4	9	ACOM	IV, V	4 × 10	Yes	RA1, LA1	No	Coiling
4/5	14	Basilar	III, I	4 × 15	No	RCa-T, RA1, RM1, RM2, LCa-T, LA1, LA2	No	Coiling
5/6	8	PICA	IV, V	4 × 20	Yes	RCa-T, RM1	No	Coiling
5/7	11	PICA	IV, V	4 × 20	Yes	LCa-T, LM1, LM2	No	Coiling
6/8	7	ACI	IV, I	4 × 10	Yes	LCa-T, LM1, LM2, RCa-T	No	Coiling
6/9	9	ACI	IV, I	4 × 10	Yes	BA	No	Coiling
6/10	10	ACI	IV, I	4 × 15	Yes	LCI	No	Coiling
6/11	13	ACI	IV, I	4 × 15	Yes	LM1, RA1	No	Coiling
7/12	9	ACOM	IV, V	4 × 15	No	LM1	No	Coiling
8/13	16	Basilar	IV, III	4 × 10	Yes	RCa-T, RM1	No	Coiling
9/14	No depiction of aneurysm	–	III, I	4 × 20	Yes	LCa-T, LM1, RCa-T, RM1	No	–
10/15	8	ACI	IV, IV	4 × 15	Yes	LCa-T, LM1	No	Clipping
11/16	7	RV	IV, III	4 × 15	Yes	LV	No	Coiling
12/17	8	ACI	IV, II	4 × 10	Yes	RA1, RM1	No	Clipping
13/18	11	ACOM	IV, I	4 × 20	No	RM1, LM1, LA1	No	Clipping
14/19	8	MCA	IV, III	4 × 10	Yes	RCa-T, RM1, LCa-T, LM1	No	Clipping
15/20	10	MCA	IV, IV	4 × 15	No	RM1, RCI, LM1	No	Clipping
16/21	6	Basilar	IV, V	4 × 15	No	LM1, LA1, RM1	No	Clipping
17/22	7	ACOM	IV, II	4 × 20	Yes	LM1, LA1	No	Coiling

SAH Fisher grade III was depicted in four patients and in thirteen patients (18 cases) grade IV SAH. Five patients (8 cases) suffered from SAH Hunt and Hess grade I, two patients (2 cases) from grade II, three patients (3 cases) from grade III, three (3) from grade IV and in four patients an SAH Hunt und Hess (6) grade V was diagnosed.

Ten patients (14 cases) were treated by a neuroradiologist of our institute with coil embolization. In six patients the ruptured aneurysm was clipped. In 1 case, clipping was performed after incomplete coil embolization.

The TBA was performed on median 8th day (IQR 7–10.25) after aneurysm rupture. We always used the Scepter C balloon catheter, which is provided in 3 sizes. We used 11 times the 4 × 15 mm balloon catheter, 7 times the 4 × 10 mm and 6 times the 4 × 20 mm. The distal internal carotid artery (incl. Carotid T) was dilated in 13 cases, the A1 segment in 9 cases, the M1 segment in 21 cases, the M2 segment in 3 cases, the A2 segment in 1 case, the distal vertebral artery in 3 cases and the basilar artery in 4 cases.

In 15 out of 22 cases, we decided on the intra-arterial use of a dihydropyridine calcium channel blocker (nimodipine) and in 1 case, opium alkaloid antispasmodic drug (papaverine) was administered. Intra-arterial doses ranged from 1.8 to 20.0 mg per treatment session.

There was a decrease of the percental temporal difference between distal vessels and the proximal reference vessel from a median of 25% (IQR 14–42) prior to dilatation, to 13% (IQR 0–22) after treatment of the vasospasm. Using the Wilcoxon-test, we noticed a statistically significant reduction of the temporal difference for the anterior circulation at proximal and at distal vessels (A1-segment *P* < 0.001, M1-segment *P* < 0.001, A2-segment *P* < 0.001, M3-segment *P* = 0.002). The statistical analysis of the hemodynamic values of the posterior circulation did not show any significant reduction (P = 0.54); however, all flow parameters showed a mild to moderate reduction of the percental temporal difference between the reference vessel and the vessel of choice after the TBA, ranging from a median of 34% (SD 14) to a median of 28% (IQR 16–35).

In 14 cases, we were able to compare the TCD values before and after the intervention. We observed a statistical significant decrease of the medial TCD values (P < 0.001, Wilcoxon-test). In 7 cases we extubated the patients in the angiography suite and in 5 of them we observed a postinterventional improvement of neurological deficits. That was considered as clinical improvement due to angioplasty. In the other 15 cases the patients remained intubated (Table 2).

**Table 2:** Clinical improvement and follow-up of patient’s outcome

Pt/Case	Deterioration or deficit prior to endovascular treatment	Clinical improvement	mRS discharge	mRS Follow-up after 3 M
1/1	Intubated	N/A	6	
2/2	Right hemiparesis	Minimal improvement	0	N/A
3/3	Intubated	N/A	3	
3/4	Intubated	N/A	3	
4/5	Left hemiparesis	Minimal improvement	3	3
5/6	Intubated	N/A	1	1
5/7	Intubated	N/A	1	1
6/8	Right arm paresis and aphasia	Aphasia completely resolved, moderate improvement of paresis	1	0
6/9	Aphasia	Aphasia completely resolved	1	0
6/10	Motoric aphasia and right hemiparesis	Moderate improvement of aphasia and hemiparesis	1	0
6/11	Aphasia, hemianopsia and right hemiparesis	No improvement	1	0
7/12	Intubated	N/A	5	N/A
8/13	Intubated	N/A	1	1
9/14	Intubated	N/A	1	1
10/15	Intubated	N/A	2	1
11/16	Intubated	N/A	1	0
12/17	Intubated	N/A	5	N/A
13/18	Disorientation and somnolence	No improvement	0	0
14/19	Intubated	N/A	4	4
15/20	Intubated	N/A	3	1
16/21	Intubated	N/A	6	
17/22	Intubated	N/A	4	3

## Discussion

The delayed cerebral ischemia (DCI) is one of leading causes of mortality or morbidity in patients with ruptured cerebral aneurysms and consecutive SAH. Due to the improvement of the survival among hospitalized patients, mortality has halved in the last two decades; however, delayed cerebral ischemia remains one of the leading preventable reasons of poor outcome [20–23].

The endovascular treatment of vasospasms includes pharmacological and mechanical dilatation. Intraarterial infusion of papaverine, nimodipine, nicardipine or verapamil has been used with various outcomes [24]. Their limitation is the short duration of benefit. A moderate clinical improvement, a reversal of delayed ischemic neurological deficits and an angiographic improvement as high as 100% has been reported after a TBA (Table 3) [24–26].

**Table 3:** Comparison to other series regarding the endovascular treatment, the angiographic and the clinical improvement, and the favourable outcome. Comparison to other series concerning primary and secondary endpoints

Series	No. Of patients	Endovascular Treatment	Angiographic improvement	Clinical improvement	Favourable outcome (mRS 0–2)
Bejjani et al., 1998 [15]	31	TBA +/− papavarine	N/A	72%	80%
Coyne et al., 1994 [14]	13	TBA	N/A	31	38
Eskridge et al., 1994 [13]	50	TBA	N/A	61	N/A
Fujii et al., 1995 [16]	19	TBA	94	63	89
Polin et al., 2000	38	TBA +/− papaverine	82	29	53
Jestaedt et al., 2008 [25]	38	TBA	100	N/A	21
Hoh and Ogilvy 2005 [26]	530	TBA	N/A	62	N/A
Jun et al., 2010	60	TBA +/−verapamil	100	N/A	61 (the only IA group incl.)
Biondi et al., 2004	25	Nimodipine	42	76	72
Feng et al., 2002	29	Verapamil	34	29	N/A
Badjatia et al., 2004	18	Nicardipine	100	42	N/A
Our study	17	TBA +/− papaverine or nimodipine	100	23	53

Although it is considered that the angioplasty is only effective at the segment of the vessel which was dilated, our results of iFlow values showed that there was a statistical significant augmentation of the hemodynamic parameters in proximal dilated arteries as well as in their distal segments. Some authors noticed that the recurrence of a symptomatic vasospasm or the depiction of vasospasm in an adjacent, distal arterial segment could be also possible [1, 27, 28]. In our collective, only two patients with SAH Fisher grade III underwent further angioplasty of more distal segment of the same vessel; nevertheless, we observed no recurrence of vasospasm of the already dilated segments or of adjacent segments. (Figs. 1 and 2).

**Fig. 3:**
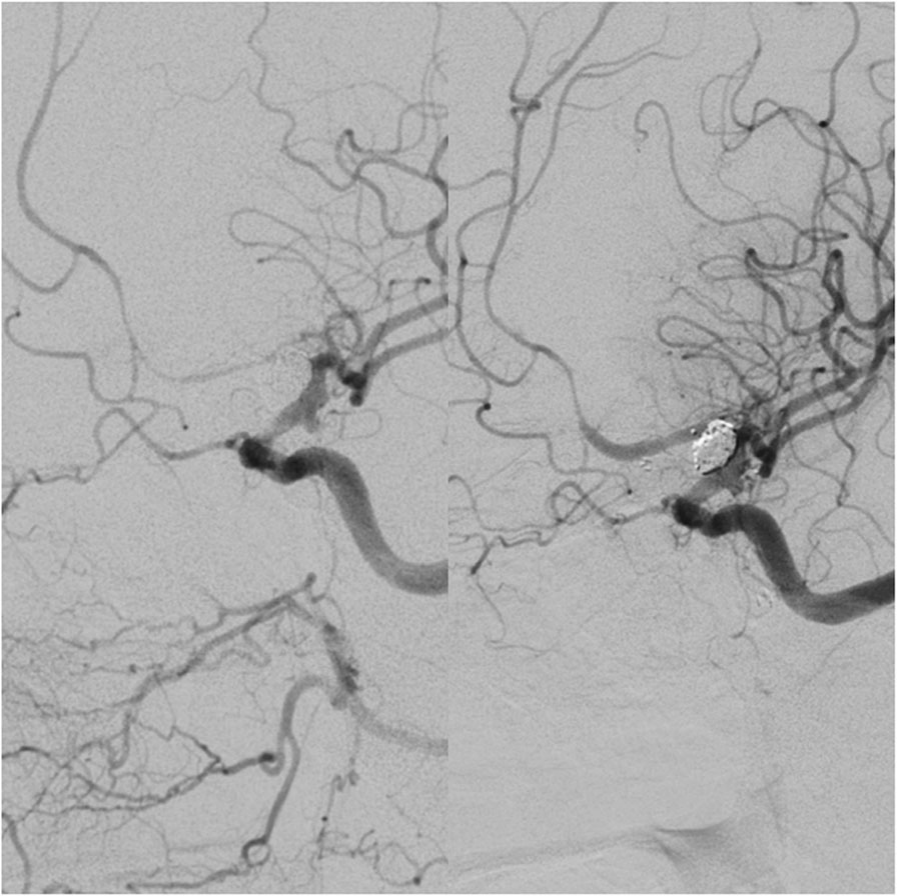
Angiographic images of a 50-year-old female patient before and after the angioplasty of the left A2 segment with notable normalization of the width of the vessel

We noticed no complications related to the use of the Scepter C balloon catheter, which is in line with findings of others [29]. Nevertheless, the comparison with the initial angiographic imaging for the estimation of the real vessel size is very important to prevent a rupture or perforation due to overinflation. This balloon catheter showed improved feasibility and trackability compared to the commonly used single-lumen over the wire balloon catheters. We could reach and treat the affected vessels in all cases. The angioplasty of the M2 or A2 segment was also possible with a 0.014 in. hydrophilic microwire (Fig. 3). This catheter is provided in 3 sizes with a constant diameter of 4 mm. A smaller diameter (e.g. 3 mm) would be even better in some cases.

The syngo iFlow software is not widely used in the clinical praxis. It provides the visualization of a complete DSA run in a color-coded single image in push-button approach (Figs. 1 and 2). Clinicians can evaluate the inflow and outflow of contrast agent in regions of interest. Measures with this software are feasible, easy, quick and reliable and offer an immediate evaluation of results of the angioplasty.

There are no guidelines for the interventional treatment of cerebral vasospasms in the literature [30]. In our experience, the intra-arterial treatment of SAH-related vasospasms seems safe and efficient. In many cases, TBA is performed after the detection of vasospasm-related infarction or severe stenosis of proximal arteries. It remains to be proved with a randomized prospective trial, if TBA should be applied earlier for the treatment of vasospasms.

The primary limitation of our study is the retrospective character and the highly selected group of patients. Another limitation is the absence of an independent core lab. We extracted data from a prospectively collected database and consecutive patients were included in our analysis. All interventions were performed only by experienced neuroradiologists. The fact that we did not observe any complications does not mean that the use of this catheter is accompanied with no risk.

## Conclusions

The aim of this retrospective study is to report our experience with the Scepter C balloon-catheter for the treatment of cerebral vasospasms after SAH and the postprocedural result evaluation with the above-mentioned software. This is the first study, which confirms that the angioplasty of proximal arteries of circle of Willis is effective also at the peripheral branches. The use of the above-mentioned balloon catheter showed improved feasibility and trackability and was not associated with complications. Additionally, we find the described software useful and reliable for the purposes of decision making. Since 2015, we use it periinterventional during a TBA to evaluate the flow of proximal and distal arteries after the angioplasty.

## Data Availability

The datasets used and/or analyzed during the current study are available from the corresponding author on reasonable request.

## Abbreviations

ACA: Anterior cerebral artery
ACM: Middle cerebral artery
BFV: Blood flow velocity
CTA: Computed tomography angiography
CTP: Computed tomography perfusion
DCI: Delayed cerebral ischemia
DSA: Digital subtraction angiography
ROI: Region of interest
SAH: Subarachnoid hemorrhage
TBA: Transluminal balloon angioplasty
TCD: Transcranial doppler sonography
